# Evaluation of a Scalable Information Analytics System for Enhanced Situational Awareness in Mass Casualty Events

**DOI:** 10.1155/2016/9362067

**Published:** 2016-06-28

**Authors:** Aura Ganz, James M. Schafer, Zhuorui Yang, Jun Yi, Graydon Lord, Gregory Ciottone

**Affiliations:** ^1^Electrical and Computer Engineering Department, University of Massachusetts Amherst, Amherst, MA 01003, USA; ^2^Intermedix Corporation, 1800 S. Bell Street, Suite 210, Arlington, VA 22202, USA; ^3^Disaster Preparedness Program, Harvard Humanitarian Initiative, Harvard University, Boston, MA 02215, USA

## Abstract

We investigate the utility of DIORAMA-II system which provides enhanced situational awareness within a disaster scene by using real-time visual analytics tools and a collaboration platform between the incident commander and the emergency responders. Our trials were conducted in different geographical areas (feature-rich and featureless regions) and in different lighting conditions (daytime and nighttime). DIORAMA-II obtained considerable time gain in efficiency compared to conventional paper based systems. DIORAMA-II time gain was reflected in reduction of both average triage time per patient (up to 34.3% average triage time reduction per patient) and average transport time per patient (up to 76.3% average transport time reduction per red patient and up to 66.3% average transport time reduction per yellow patient). In addition, DIORAMA-II ensured that no patients were left behind or transported in the incorrect order compared to the conventional method which resulted in patients being left behind and transported in the incorrect order.

## 1. Overview

In March 2011, a large earthquake hit the coast of Japan and triggered a powerful tsunami. The incident caused more than 15,000 deaths and 2,000 people were missing. Mass casualty incidents (MCIs) caused by natural disasters such as earthquakes and floods have caught the world attention during the past few years. Disaster is defined as “a serious disruption of the functioning of a society, causing widespread human, material or environmental losses which exceed the ability of the affected society to cope using only its own resources” by the United Nations Disaster Management Training Program.

After a disaster occurs Emergency Medical Services (EMS) play an important role by providing effective, responsible prehospital care. Their performance influences the chance of survival among patients.

The current conventional methods for triage and transport include the use of paper triage tags as well as radio based communication between the incident commander and emergency responders. In the conventional system the incident commander and responders have limited situational awareness of the incident as well as limited knowledge of the progression of triage and transport operations.

In [1] the authors introduce DistressNet, which provides smart patient detection under rubble and digitized building information. There are no visualization tools for the incident commander or any patients tracking in real time. No trial results are reported.

The Smart Systems Research Laboratory (SSRL) responder tool [2] provides location of the responders as well as the ability for the responders to report free-form text comments and digital images enhancing the situational awareness. The incident commander has no means to interact with the responders and has no ability to provide real-time tracking of patients.

In [3] the authors describe a decision support system FRIEDAA that enables responders to collect spatial-temporal information about the patients. Moreover, a visualization tool displayed on a large tactile screen is developed for the incident commander to track the operational progress. FRIEDAA lacks the ability to keep track of the patients' movement continuously.

In [4, 5] the authors focus on reporting building information in disaster scenario which can help the incident commander evaluate the buildings condition.

DIORAMA system which we introduced in [6, 7] provides a comprehensive situational awareness and communication platform between the incident commander and the emergency responders and will significantly enhance the effectiveness of the response. Moreover, the system integrates seamlessly with the emergency responders current triage and transport procedures. In Section 2 we describe DIORAMA-II cloud-based server structure, the incident commander and emergency responder software tools, and the recurrent localization algorithm that enables us to localize patients in real time using a mobile anchoring system.

In this paper we focus on studying DIORAMA-II utility compared to conventional paper based triage and transport methods. We conducted trials in different geographical areas (feature-rich and featureless areas) and different lighting conditions (during daytime and nighttime). The quantitative performance metrics we use to evaluate DIORMA-II utility include the average triage time per patient, average transport time per patient, number of patients left behind in the field, and transport order. In addition, we also administer a qualitative questionnaire to the emergency responders that helps us understand DIORAMA-II usability and potential adoption by the emergency responder community.  Section 3 details the trials conducted as well as the quantitative and qualitative performance results.  Section 4 concludes the paper.

## 2. DIORAMA-II Architecture

**Figure 1:**
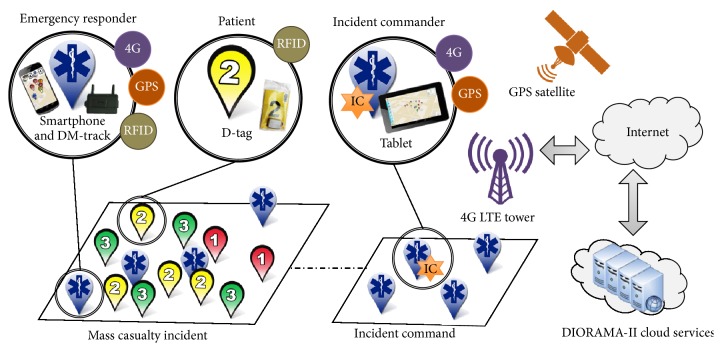
DIORAMA-II architecture.

DIORAMA-II architecture shown in Figure 1 was introduced in [7]. The responders carry an active RFID reader denoted as DM-track and a Smartphone. The responders tag each patient with an active RFID tag (D-tag) along with a paper triage tag. The DM-track collects received signal strength indicator (RSSI) readings from the D-tags which represent measurements of the power present in received radio signals. These readings are relayed to the server through the Smartphone. DIORAMA-II server which is implemented in the cloud (1) hosts DIORAMA-II services, (2) hosts the localization engine which calculates the location of all patients and responders, and (3) maintains the database that receives database transactions from the DIORAMA-II service to retrieve, update, insert, and delete DIORAMA-II related information.

### 2.1. DIORAMA-II Cloud Services

A cloud-based approach in DIORAMA-II brings several important advantages.

*Scalability.* Incidents can vary significantly in size with respect to geographical region and the number of patients and responders. This is accomplished by partitioning the DIORAMA-II server architecture components into virtual machines that have discrete roles. Therefore as the load increases on a virtual machine with a specific role, another identical virtual machine will switch on to assist and alleviate the load. The ability to divide the load across multiple virtual machines allows DIORAMA-II system with the appropriate optimization to scale to an incident of any size.

*Resource Savings.* Most day-to-day incidents are relatively small but there is always the potential for a large-scale MCI. With cloud architecture only the virtual machines utilized are being paid for.

*Reliability.* It is important that DIORAMA-II is reliable throughout the entire duration of the incident. If there were to be a fatal error in one of the DIORAMA-II virtual machines, another virtual machine would switch on and take in all the load from the virtual machine with the fatal error, allowing the DIORAMA-II server to continue running.

*Redundancy.* When a large-scale disaster occurs in a specific region, it is likely that the infrastructure itself is damaged. If the DIORAMA-II server was to be hosted geographically locally to the location in which the disaster occurs this could result in the services becoming unavailable because the server can be damaged. A cloud-based architecture allows for georedundancy allowing virtual machines to be placed in locations throughout the world and therefore if one cloud site was to become compromised due to a disaster, one of the unaffected locations could be utilized.

DIORAMA-II server which is deployed in the cloud includes three components. 

(*1) MS SQL Database Virtual Machine (VM).* The MS SQL Database VM is where all DIORAMA data is persistently stored. Through Microsoft Azure Cloud [8] we have local and geographical redundancy.

(*2) DIORAMA Services VM.* DIORAMA Services VM is built using Microsoft Azure App Services [8]. The DIORAMA Services VM is the universal communication interface for the client devices and localization engine VM with the database VM. The App Services VMs by design can automatically scale out the service based on load or server fault, but for DIORAMA system a similar georedundant approach has been created where if the client or localization engine were unable to perform a retrieve or send operation with DIORAMA Services VM, they would then try the georedundant VM. We have simulated a failure in the DIORAMA Services VM by turning off the local VM. When the clients try to retrieve or send operation to the local VM they will have a timeout after five seconds. After three failed timeouts the client will then perform the same operation on the georedundant VM. If this is the first request made to the VM there is a cold start delay on average of 12 seconds. This means the first client that switches to a cold VM will have an average delay of 27 seconds, and every client afterwards would take 15 seconds; however there is no data lost and after the client reconnects to the georedundant service the system continues to run as normal.

(*3) Localization Engine VM.* The Localization Engine VM is a classic VM that runs Microsoft Windows Server 2012 R2. Unlike the two prior VMs detailed above, the majority of the classic VM configuration, including scaling to load and recovery of the VM, is self-managed. Currently in order to scale out the system, identical images of the VM must be started manually. We plan to enhance the localization engine by automating this process. However, in the event of a failure of this VM, all of the data needed to calculate the localization is still being stored in the database. Therefore, after a fresh VM is manually started it will resume calculating positions of patients in the field.

### 2.2. DIORAMA-II Applications

DIORAMA-II system includes two main applications: the incident commander application and the responder application.

This incident commander and responder applications which we introduced in [7, 9, 10] display in real time the icon view of the incident overlaid on Google Maps. The icons represent the points of interest (patients, responders, etc.) of the incident. In addition, the responder application includes an augmented reality (AR) view of the patients overlaid on the camera view. AR view will enable the responder to easily find the patient that should be evacuated even if the patient is hidden by obstacles such as debris or buildings.

### 2.3. DIORAMA-II Localization Engine

Various RFID localization algorithms and applications have been developed in the last decade. According to [11], such algorithms can be categorized into multilateration, Bayesian inference, nearest neighbor, proximity, and kernel-based learned methods. More details about RFID-based localization algorithms can be found in [11–13].

The localization algorithm first introduced in [7] is an original contribution since it is tailored to the portable and cost-effective hardware that we use such as the Smartphones and active RFID technology (tags and readers). Moreover, our approach does not require any fixed infrastructure and no calibration of the devices, fact that translates into no deployment overhead (in time and cost). Moreover, our localization algorithm considers the triage and evacuation application which is designed to assist. For example, we use an iterative tracking algorithm which assumes that most of the triaged patients stay still (even the moving patients will eventually stop moving), fact that enables us to accumulate more RSSI readings for improving the localization accuracy.

To localize and update the patients' locations, the localization engine processes the information in the following steps.

**Step 1 .**

Acquire all the readings information from each responder in the past 10 seconds. Each reading includes RSSIs received from nearby D-tags, responder ID, patient ID, timestamp, and GPS coordinates. We know for each responder his/her GPS coordinates as well as the RSSI received from the patients in the vicinity.

**Step 2 .**

We define a cost map (100 ft by 100 ft) centered at the GPS coordinates of the responder. The map includes 10,000 grids of size 1 ft by 1 ft. Calculate the distance between the coordinates of each grid on the cost map  and the GPS coordinates of the responder. Using the signal strength attenuation model we introduced in [7], we calculate the expected RSSI in each grid and define the following cost function for each grid:

(1) $$\begin{array}{c} {\text{Cost}}_{\left(x,y,t_{k}\right)}=\overset{i=k}{\underset{i=1}{\sum }}{\left|\;S_{\text{expected}\left(x,y,t_{i}\right)}-S_{\text{measured}\left(x,y,t_{i}\right)}\;\right|}^{2}, \end{array}$$

where Cost_(*x*, *y*, *t*_*k*_)_ is the cost value on grid (*x*, *y*) up to time *t*_*k*_, *S*_expected(*x*, *y*, *t*_*i*_)_ is the expected RSSI on grid (*x*, *y*) at time *t*_*i*_, and *S*_measured(*x*, *y*, *t*_*i*_)_ is the measured RSSI on grid (*x*, *y*) at time *t*_*i*_.

**Step 3 .**

The algorithm computes the location in the sampling area with the minimum cost as the estimated location of the patient. With the cost function defined above, the location can be estimated as

(2) $$\begin{array}{c} {\left(\;\hat{x},\hat{y}\;\right)}_{t}=\underset{\left(x,y,t\right)}{\mathrm{argmin}\;}\text{Cost}\left(\;x,y,t\;\right). \end{array}$$

It is important to mention that Steps 2 and 3 are performed for each patient.

The opportunistic localization algorithm average accuracy is less than 6 meters which was achieved through extensive testing in real deployments. We investigated how the localization accuracy is influenced by parameters such as the number of RFID readers (i.e., the number of responders) and the number of RFID tags per patient. The size of the localization area was not considered since the algorithm is inherently independent of the area.

We observed, contrary to our intuition, that there is no relationship between the number of the tags on each patient and the localization accuracy. This is due to the fact that we have enough diversity of readings from tags since we have mobile localization anchors; that is, the responders who are equipped with the readers are moving around. Therefore, due to cost considerations we have chosen to use one tag per patient.

As expected the localization accuracy improves as the number of responders (i.e., readers) in the vicinity of the patients increases. This is due to the fact that our algorithm is opportunistic; that is, the larger the number of mobile anchors the higher the accuracy. However, in our application we do not have influence on this parameter since we cannot control the responders movements.

We would like to emphasize that the localization accuracy is not the main objective of the system since absolute coordinates of the patient are very difficult to understand on a user interface. As determined by our trials the localization accuracy obtained by the opportunistic localization algorithm (average of 6 meters) is acceptable. Obviously if we have a fixed system which requires careful calibration we can obtain better localization accuracy. One of the most important design guidelines in our system is the localization system mobility and the fact that we do not need to calibrate the system when we arrive on the mass casualty site.

### 2.4. Discussion about Battery Usage

In this subsection we discuss battery usage and battery life of the Smartphone used by the responders. The DIORAMA responder application uses GPS, Bluetooth, data, camera, RFID, and display.

We conducted a number of battery utilization tests on the DIORAMA responder application using Samsung Note 5 with a 3020 mAh battery. In all tests the data connection, GPS, and Bluetooth connection to the active RFID reader are on and the screen brightness was set to 70%.

These utilization tests fall into the following three usage modes:

1. Main screen: the responder uses the phone for the triage procedure using RFID tags as well as the bird's-eye GUI where the responder can view the surrounding patients and responders at the incident. Using this mode the battery drained in 240 minutes of continuous usage.
2. Augmented reality: the responder uses AR to find patients. Note that in this mode we use the camera. The battery drained in 169 minutes of continuous usage.
3. Standby: the phone screen is off but data is still being collected and sent to the server. The battery drained in 309 minutes of continuous usage.

As expected, the AR mode utilizes the most power due to the continuous use of the camera.

In reality the responder will use a combination of the three modes. Assuming the responder will use the modes equally, the Smartphone will last about 4 hours. These results also reflect actual usage by EMTs in our trials.

Note that the longevity of the battery may differ for various phones due to different battery specifications and power usage of the GPS, camera, and display. To prolong the battery life of the Smartphone during large MCI we recommend the use of an extended battery-pack.

## 3. DIORAMA-II Simulation Trials

We measure the improvements of the DIORAMA-II system compared to the conventional system in terms of average primary triage time per patient, average transport time per patient, transport order, and transport completeness. Transport is defined as the transportation of patients from the location of injury to the treatment area.

Within the scope of the trials we have emergency responders and patients. The emergency responders role was performed by certified EMTs from the local University of Massachusetts Amherst Emergency Medical Services unit. The emergency responders prioritize the transport of patients in the mass casualty incident (MCI) hot zone through the use of the triage process. After the patients are triaged the emergency responders transport each patient from the hot zone in order of priority to the treatment area. The emergency responders are assigned the following roles: incident commander (IC), triage emergency responder, and transport emergency responder.

Each patient (represented by a human subject or a cone) was assigned a triage tag denoting his priority and was placed in a designated location throughout the hot zone.

Each trial is composed of a triage and transport phase.

In the triage phase the emergency responders under the direction of the incident commander search for patients within the incident. When the responder identifies an injured patient they perform primary triage which is a quick assessment of the patient's criticality based on any immediate life-threatening injuries. To ensure that the responder assigns a consistent priority to the same patient (human or cone) in both the conventional and DIORAMA-II trials, each patient is assigned a predetermined priority cue card. The emergency responder assigns the triage priority according to the cue card. The phase ends once the incident commander believes the emergency response has covered the entire area at which point the total time for triage is recorded. In the conventional method the responders tag the patient with a paper tag and report to the incident commander the priority level and estimated relative location of each triaged patient. In the DIORAMA-II trials the responder tags the patients with a D-tag. DIORAMA-II applications collect the patients' priority and location information and display it to both the incident commander and emergency responders.

In the transport phase the incident commander directs the emergency responders to transport patients from their location of injury to the treatment area. Patients are transported in order of their triage priority, first red patients followed by yellow. When a patient is transported to the treatment area their unique ID#, triage priority, time of arrival, and ID# of the emergency responder performing the transport are recorded. During this phase the incident commander uses the information collected from the triage phase to direct the emergency responders to the triaged patients in the incident. The efficiency of the transport phase relies on accuracy of the information collected. Inaccurate information can cause the emergency responder to spend more time searching for a patient or if a patient is unaccounted for this can result in them being left injured in the field. In the conventional trial this information can be inaccurate since it is conveyed through the radio units and the disaster area may not have enough landmarks for accurate verbal description. On the other hand, DIORAMA-II system will collect accurate information implicitly and convey it in a visual form to both the incident commander and responders resulting in a significantly faster transport process.

We will compute the following quantitative performance metrics for each trial:

- *the average triage time per patient *(obtained as the total triage time divided by the number of patients triaged),
- *the average transport time per red patient *(obtained as the total transport time for red patients divided by the number of red patients transported),
- *the average transport time per yellow patient *(obtained as the total transport time for yellow patients divided by the number of yellow patients transported),
- *% time gain*: reduction of time in DIORAMA-II trial compared to conventional trial; we will discuss % time gain in both triage time and transport time,
- *the number of patients left behind *when the trial ends,
- *the transport order*: the right order transports the red patients before the yellow patients.

We conducted two sets of trials as described in Table 1.

*Set 1.* Trials were conducted in two distinct feature-rich regions (e.g., university campus) during daytime. Details are provided in Section 3.1. 

*Set 2.* Trials were conducted in a featureless open field region during nighttime. Details are provided in Section 3.2.

### 3.1. Set 1 Trials: Feature-Rich Area

**Figure 2:**
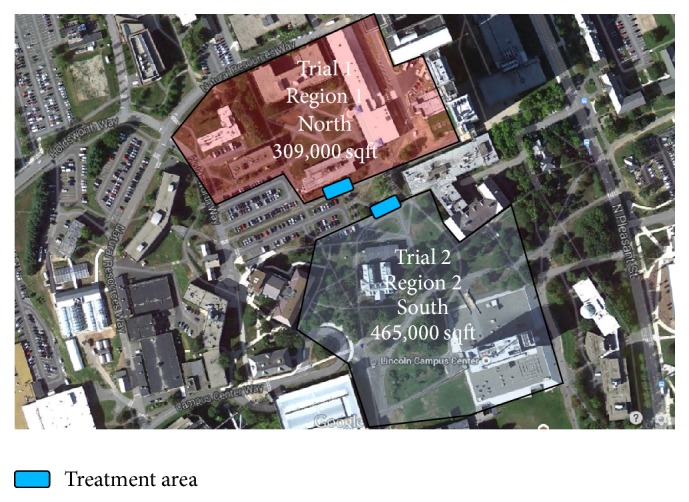
Trials 1 and 2 regions.

We conducted two simulated disasters at the University of Massachusetts Amherst campus. In each simulation we assume a tornado touches down and many students are injured. In each simulation we have two regions (see Figure 2) which differ in size, elevation, and field of view.

*Trial 1.* Trial 1 was conducted in a region of approximately 309,000 sqft that encompasses 7 academic buildings. The buildings and shrubbery within this region provide obstructions for the emergency responders both blocking their full view of the entire area and dividing the area into regions in which the responders must walk around buildings to get to a patient. 

*Trial 2.* Trial 2 was conducted in a region of approximately 465,000 sqft that encompasses 2 academic buildings. The region is mainly wide open with one building located centrally and the campus center upper pavilion which is a concrete structure elevated 25 ft higher than the rest of the region that can be accessed through concrete stairways that surround the pavilion.

#### 3.1.1. Experimental Design

**Figure 3:**
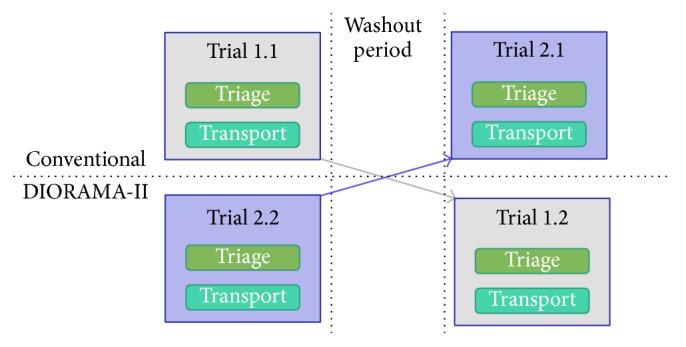
Crossover experimental design.

The trials follow a two-group crossover design (see Figure 3) which has two advantages over a noncrossover longitudinal study. First, the influence of confounding covariates is reduced. Second, optimal crossover designs are statistically efficient and so require fewer subjects than do noncrossover designs. In our simulations we use crossover design as follows: each trial is divided into two separate simulations. Each simulation has two simultaneous triage and transport trials. One trial will perform DIORAMA-II primary triage and transport while the other will perform conventional primary triage and transport. The following simulation will occur on a separate day. The emergency responders that performed a DIORAMA-II trial would then perform a conventional trial using an identical trial setup. The emergency responders that performed the conventional trial would then perform a DIORAMA-II trial with an identical trial setup. These two simulations must be separated by a period of at least one week, defined as a washout period. Since the participants will be performing the same trial setup this washout period is necessary for the participants to forget the details regarding the trial setup such as patients locations and priorities. Each emergency responder involved will perform the exact role in each simulation. In effect,* each responder serves as his/her own control*. Also, since the same responder will experience both paper tags and DIORAMA-II tags, there is no possibility of covariate imbalance.

#### 3.1.2. Quantitative Results

*(i) Primary Triage*.  Table 2 reports the average triage time per patient for Trials 1 and 2 for DIORAMA-II and conventional trials. As shown in Table 2, in Trial 1 we have 34.3% reduction in primary triage time with DIORAMA-II compared with conventional trials. In Trial 2 we have 14.7% reduction in triage time with DIORAMA-II compared with conventional trials. We obtain this significant reduction in time due to the use of the collaboration tools between the incident commander and the emergency responders. The DIORAMA-II collaboration tools display clearly to each emergency responder what their assigned area is and there is no overlap between the areas where patients have been triaged by different emergency responders. In the conventional trials we observed significant overlap between the areas where the emergency responders are looking for patients that have already been triaged.

The reason we obtained less reduction in triage time in Trial 2 is that one of the triage emergency responders did not move and did not communicate with the incident commander for a couple of minutes during DIORAMA-II Trial 2.2 (we discover this fact when we watched the video recordings of the incident). We cannot explain why this responder stalled during this time. This phenomenon highlights the necessity for adding additional features in DIORAMA-II that can monitor the movements of each emergency responder and notify the incident commander when they do not move for a specific amount of time. Such a feature is especially important because it can alert the incident commander to check if an emergency responder is injured.

*(ii) Transport*. Tables 3 and 4 include the average transport per red and yellow patient in Trials 1 and 2, respectively. From Table 3 we observe that in Trial 1 DIORAMA-II average transport per red patient was faster than conventional trials by 17% and 47.4% for yellow patients. As shown in Table 4, Trial 2 DIORAMA-II transport was faster by 30.6% and 29.1% for red and yellow patients, respectively.

We obtained a significant reduction in the average transport time per patient due to the following reasons: (1) DIORAMA-II emergency responder user interface (either map view or AR) enabled each responder to easily find the patients to be transported and (2) each transport emergency responder was assigned by the incident commander the area and the patients that need to be transported. In contrast, in conventional trials the transport emergency responders were searching for patients randomly. They did not know the patients location, their priority, nor the number of patients of each priority.

*(iii) Transport Order*. In DIORAMA-II Trial 1.2 and Trial 2.2 all patients were transported in proper order; that is, all red patients were transported before the yellow patients. However, in conventional Trial 2.1 a transport emergency responder did not transport the patients in proper order: a red patient was transported after two yellow patients.

#### 3.1.3. Qualitative Results

The qualitative results provided by the emergency responders that participated in Trials 1 and 2 are provided in Table 5. We observe that the emergency responders were very satisfied with DIORAMA-II system and found the tool useful in managing a disaster.

### 3.2. Set 2 Trials: Featureless Area

We observed during the Set 1 trials that the emergency responders had a significant familiarity with the feature-rich environment. They not only were familiar with the location where the trials took place but also received training and practice simulation on how to use DIORAMA-II in this location. For the conventional trial this gave the emergency responders the ability to relay back to the incident commander accurate locations of patients while providing their approximate location to campus landmarks. Therefore, the incident commander was able to draw up a detailed map of the patient locations and priorities. In addition, the incident commander during the transportation phase was able to relay these recorded locations to responders in the field. We hypothesized that if we put the emergency responders in an unfamiliar area that lacked landmarks we would see even more time gain in DIORAMA-II system.

In these trials we tested DIORAMA-II performance in a disaster area that has almost no landmarks.

**Figure 4:**
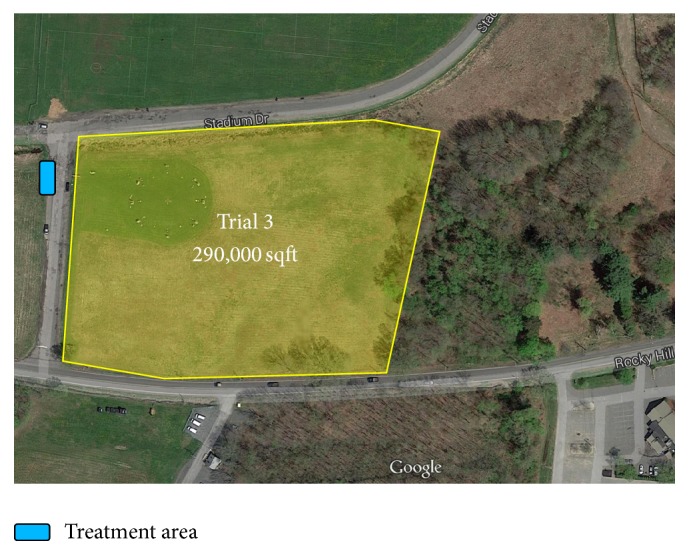
Trial 3 region.

*Trial 3.* Trial 3 was conducted in a region of approximately 290,000 sqft in size as illustrated in Figure 4. A quarter of this area contains a life-size sundial made up of various sized stones. The remaining area is a flat grass field where the grass is approximately 2 feet in height. The eastern perimeter of the field is a forest, the southern one is a road, and the northern and western sides are a stream creating a distinct boundary from the area. With the exception of the boundaries themselves and the sundial there are not too many distinguishing features in this location.

#### 3.2.1. Experimental Design

Each trial was performed twice, once for conventional method (Trial 3.1) and once for DIORAMA-II (Trial 3.2). The repeated trial was performed on a separate day, one week later, to act as a washout period in the hope that the returning emergency responders would not remember locations of patients.

Each patient, who was simulated in the field by an orange soccer cone, was assigned an envelope that contained a predetermined triage status (green, yellow, red, and dead) and if the patient is trapped. The emergency responders assign the patient the triage priority defined in the envelope.

For consistency of comparison each patient location was predetermined.

The emergency responders in both DIORAMA-II and conventional trials utilized their personal radio units that they normally would have on campus when on duty. Throughout both the DIORAMA-II and conventional trials sirens would sound as well as additional loud noises. This is to help simulate the chaotic noise found normally at an incident making it difficult for the incident commander to communicate with the response team in the field.

#### 3.2.2. Quantitative Results

*(i) Primary Triage*. In Trial 3 we have 6% per-patient triage time gain (reduction in triage time in DIORAMA-II compared to conventional method). Although this is a modest time gain this reinforces the belief made in Section 3.1.2(i) that the DIORAMA-II collaboration tools available to the emergency responders enhance the collaboration between the incident commander and the emergency responders and increase their overall efficiency.

*(ii) Transport*.  Table 6 includes the average transport per red and yellow patient in Trial 3. We observe that DIORAMA-II average transportation per patient was 76.3% and 66.3% faster than the conventional method for red and yellow patients, respectively.

By the time the transportation of the patients to the treatment area began the sun was down for both trials. There was very little visibility in the field and the responders relied on flashlights to see anything in front of them. The addition of the long grass in the field complicated identifying patients from a distance, even with the assistance of a flashlight. We observed that in conventional Trial 3.1 the emergency responders had a tremendous task searching for patients in the dark in such a large open area. The frustration could be visibly seen from the responders sweeping the area for patients while during DIORAMA-II Trial 3.2 the responders were easily directed to the approximate location of the patient using the DIORAMA-II application and were able to quickly find the patient, who was often right in front of them or a few feet away.

During conventional Trial 3.1 the incident commander was unable to build a patient layout map of the scene. The lack of landmarks and visibility made it difficult for the emergency responders performing the triage to indicate where patients were located, while during DIORAMA-II Trial 3.2 a concise map of the incident was being generated dynamically. The incident commander knew exactly how many, where, and what priority the patients were. The incident commander was then able to delegate patients for transport to the emergency responders in the field who were able to quickly find these patients and transport them to the treatment area.

*(iii) Transport Order*. In DIORAMA-II Trial 3.2 all patients were transported in proper order; that is, all red patients were transported before the yellow patients. However, in conventional Trial 3.1 after 31 minutes into the transportation phase, only 2 red patients were transported to the treatment area. The incident commander in desperation decided to move to collect yellow and red patients simultaneously due to the great length it took searching for the patients. This resulted in one transport order mistake for conventional Trial 3.1.

*(iv) Patients Left Behind*. In DIORAMA-II Trial 3.2 all patients were transported from the field to the treatment area. However, in conventional Trial 3.1 there were a total of 7 patients left behind (3 red and 4 yellow). The emergency responders in conventional Trial 3.1 had a difficulty searching for patients and were only successful at transporting 5 out of the 12 injured patients.

#### 3.2.3. Qualitative Results

The qualitative results provided by the emergency responders that participated in Trial 3 are provided in Table 7. We observe that the emergency responders were very satisfied with DIORAMA-II system and found the tool useful in managing a disaster.

### 3.3. Summary of Simulation Results

DIORAMA-II ability to provide situational awareness to the emergency responders coupled with the application's visualization tools such as AR was able to empower the emergency responders which resulted in considerable reduction in both triage time and transport time. In addition, DIORAMA-II ensured that no patients were left behind or transported in the incorrect order compared to the conventional method which resulted in patients being left behind and transported in the incorrect order. The results obtained by DIORAMA-II compared to the conventional method are summarized in Table 8.

DIORAMA-II showed the highest time gains in Trial 3 that took place at night in a featureless location. This clearly demonstrates the utility of DIORAMA-II in unfamiliar and featureless environments. It is the case in many larger incidents that emergency responders from the surrounding areas are dispatched to assist. It is unlikely that these responders are familiar with the location or received training at the location of the incident. DIORAMA-II will enable the emergency responders to effectively carry out their tasks.

## 4. Conclusions

The results show the following advantages to DIORAMA-II system compared to conventional triage:

1. DIORAMA-II obtains significant reduction of both average triage per patient and average transport time per patient.
2. DIORAMA-II obtains higher reduction in average transport time per patient in featureless areas.
3. In DIORAMA-II we do not have any patients left in the field when compared with the conventional method which has left patients in the field.
4. In DIORAMA-II we obey the transport order (first red and then yellow) as opposed to the conventional method where this order is not followed.

## Figures and Tables

**Table 1:** Trials overview.

Set 1		Set 2
Daytime		Nighttime
Feature-rich area		Featureless area
Trial 1.1: conventional	Trial 2.1: conventional	Trial 3.1: conventional
Trial 1.2: DIORAMA-II	Trial 2.2: DIORAMA-II	Trial 3.2: DIORAMA-II area

*24 patients*	*25 patients*	*27 patients*
(i) 6 red	(i) 5 red	(i) 7 red
(ii) 6 yellow	(ii) 6 yellow	(ii) 5 yellow
(iii) 10 green	(iii) 7 green	(iii) 8 green
(iv) 2 black	(iv) 7 black	(iv) 7 black
*Emergency response *	*Emergency response*	*Emergency response*
(i) 2 primary triage	(i) 2 primary triage	(i) 2 primary triage
(ii) 2 transport to treatment	(ii) 2 transport to treatment	(ii) 2 transport to treatment
(iii) 1 IC	(iii) 1 IC	(iii) 1 IC

**Table 2:** Average triage time per patient in Trials 1 and 2.

	DIORAMA-II (mm:ss)	Conventional (mm:ss)	% time gain for DIORAMA-II
Avg triage time per patient in Trial 1	*Trial 1.2 *00:44	*Trial 1.1 *01:07	34.3%
Avg triage time per patient in Trial 2	*Trial 2.2 *00:53	*Trial 2.1 *01:02	14.7%

**Table 3:** Average transport time per patient in Trial 1.

	DIORAMA-II (Trial 1.2) (mm:ss)	Conventional (Trial 1.1) (mm:ss)	% time gain for DIORAMA-II
Avg transport time per red patient	03:30	04:13	17%
Avg transport time per yellow patient	02:16	04:19	47.4%

**Table 4:** Average transport time per patient in Trial 2.

	DIORAMA-II (Trial 2.2) (mm:ss)	Conventional (Trial 2.1) (mm:ss)	% time gain for DIORAMA-II
Avg transport time per red patient	03:55	05:39	30.6%
Avg transport time per yellow patient	02:22	03:20	29.1%

**Table 5:** Trials 1 and 2 qualitative results.

Feedback for user interface	Not satisfactory	Satisfactory	Excellent	
Location of patients	0	0	8	
Summary of patients of each triage color	0	0	8	
Summary of transported patients	0	2	6	

	Yes	No	Maybe	N/A

Did you feel like you were in control of the transport process?	8	0	0	0
Remote use of the tool (you can be located off-site to use it)	8	0	0	0
Tool is useful in managing extrication and disposition of patients	8	0	0	0
Tool is helpful in better managing resources	8	0	0	0

**Table 6:** Average transport time per patient in Trial 3.

	DIORAMA-II (Trial 3.2) (mm:ss)	Conventional (Trial 3.1) (mm:ss)	% time gain for DIORAMA-II
Avg transport time per red patient	02:43	11:29	76.3%
Avg transport time per yellow patient	01:38	04:50	66.3%

**Table 7:** Trial 3 qualitative results.

Feedback for user interface	Not satisfactory	Satisfactory	Excellent	
Location of patients	0	0	5	
Summary of patients of each triage color	0	0	5	
Summary of transported patients	0	0	5	

	Yes	No	Maybe	N/A

Did you feel like you were in control of the transport process?	3	0	0	2
Remote use of the tool (you can be located off-site to use it)	5	0	0	0
Tool is useful in managing extrication and disposition of patients	5	0	0	0
Tool is helpful in better managing resources	3	0	2	0

**Table 8:** Summary of results obtained in each trial.

	Trial 1	Trial 2	Trial 3
% time gain per patient triaged	34.3%	14.7%	6%
% time gain per red patient transported	17%	30.6%	76.3%
% time gain per yellow patient transported	47.4%	29.1%	66.3%
# of transport order errors	Trial 1.1: 0 Trial 1.2: 0	Trial 2.1: 2 Trial 2.2: 0	Trial 3.1: 1 Trial 3.2: 0
# of patients left behind	Trial 1.1: 0 Trial 1.2: 0	Trial 2.1: 0 Trial 2.2: 0	Trial 3.1: 7 Trial 3.2: 0
